# Water adsorption on a model silicate surface: wollastonite (100)

**DOI:** 10.1039/d6nr01063f

**Published:** 2026-05-16

**Authors:** Luca Lezuo, Andrea Conti, Alexander Hoheneder, Elena Vaníčková, Domitilla Alessandra Aloi, Rainer Abart, Florian Mittendorfer, Michael Schmid, Ulrike Diebold, Giada Franceschi

**Affiliations:** a Institute of Applied Physics, TU Wien 1040 Vienna Austria franceschi@iap.tuwien.ac.at; b Central European Institute of Technology, Brno University of Technology 61200 Brno Czech Republic; c Department of Lithospheric Research, Universität Wien 1090 Vienna Austria

## Abstract

Water adsorption on silicate surfaces is a critical yet poorly understood process relevant to, *e.g.*, mineral weathering and cement hydration. This study investigates the structure of water overlayers on a model calcium silicate, the lowest-energy (100) surface of wollastonite (CaSiO_3_). It combines atomically resolved non-contact atomic force microscopy (nc-AFM), acquired with qPlus sensors and functionalized tips in ultrahigh vacuum (UHV), with density functional theory (DFT) calculations employing the metaGGA r^2^SCAN + rVV10 functional. Adding incremental doses of water to the sample at cryogenic temperatures produces distinct structures governed by the competition between water–surface and water–water interactions. With two water molecules per surface unit cell, water–surface interactions dominate: In line with previous theoretical predictions, adsorbates follow the surface lattice. As the coverage increases, intermolecular hydrogen bonding competes with bonding to the surface, leading to the emergence of complex, coexisting patterns. While their small energy differences prevent an unambiguous identification of the most stable structure by DFT, the experimentally observed symmetries help constrain plausible structural models. Above a critical density of four water molecules per unit cell, water–water interactions prevail, and water clusters are formed. The results provide an atomic-scale framework for understanding water interactions with calcium silicate surfaces.

## Abstract 2

Atomic-scale nc-AFM and DFT reveal water adsorption mechanisms on a model calcium silicate relevant to cement chemistry.

## Graphical abstract

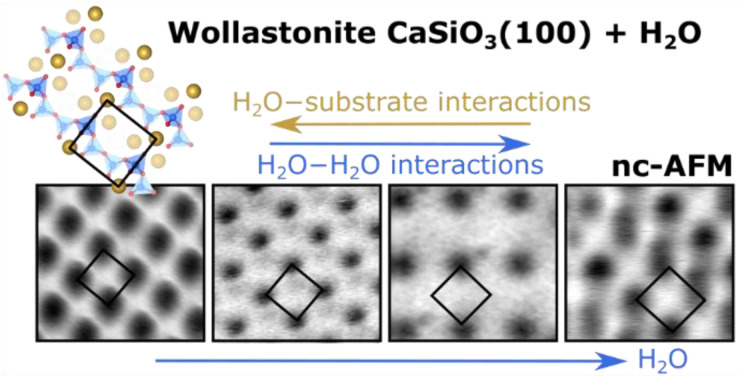

## Introduction

At the molecular scale, the behavior of water at solid interfaces is governed by a delicate balance between water–water cohesion and water–surface adhesion. This competition is central to processes as diverse as ice nucleation, surface wetting, catalysis, rock weathering, and cement hydration.^1–5^ It determines whether water forms bulk-like, hydrogen-bonded (H-bonded) clusters or adsorption structures with a strong relationship to the underlying lattice.^6^ The surface science approach can provide atomic-scale insights into this competition by studying water on idealized, single-crystalline surfaces in ultrahigh vacuum (UHV) and from cryogenic temperatures to room temperature. While this strategy has led to a deep understanding of water adsorption on metals^7–9^ and selected oxide systems,^10–16^ the interaction of water with natural silicate minerals remains less explored. Yet, water–silicate interactions underpin many key natural and technological processes; among others, the stabilization of concrete.^5^

**Fig. 1:**
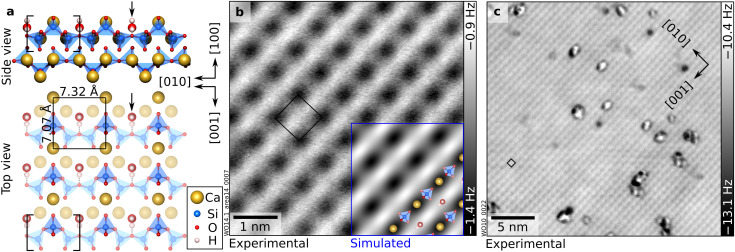
As-cleaved, hydrated wollastonite (100): one H_2_O molecule per unit cell. (a) DFT-optimized surface structure of wollastonite (100) with one H_2_O per (1 × 1) surface unit cell adsorbed in the “nested” configuration (black arrow). Brackets indicate the *dreierketten* repeat unit (one upper and two lower silica tetrahedra) of the silicate chain running along the [010] direction. (b and c) 5.3 × 5.3 nm^2^ and 27 × 27 nm^2^ nc-AFM images of the UHV-cleaved surface that has reacted with H_2_O at room temperature. Dark grey indicates attractive tip–surface interaction, while in the light-gray regions, tip–sample interaction is weak. The inset in panel (b) shows an AFM simulation derived from the DFT model on the left-hand side (tip–sample distance of 5.7 Å). AFM parameters: (b) *A* = 700 pm, *V*_s_ = −2.5 V, Cu-terminated tip. (c) *A* = 550 pm, *V*_s_ = −10 V, wollastonite-modified tip. (1 × 1) unit cells are marked in black.

The main binding material of concrete is cement, a hydrated paste rich in C–S–H (calcium–silicate–hydrate). C–S–H is a complex, heterogeneous structure built from “*dreierketten*” silicate chains (repeating units of three SiO_4_ tetrahedra), interlayer calcium, and molecular water.^5^ Because water actively participates in both the formation and the final structure of C–S–H, its role has been investigated extensively through simulations, including studies of C–S–H nucleation,^17^ the distribution and reactivity of short silicate chains,^18^ and the nature of interfacial water on C–S–H surfaces.^19^ However, experimental benchmarks remain scarce: macroscopic observables such as the Ca : Si ratio, density, and mechanical properties provide only indirect information about the molecular-scale structure of C–S–H and its hydration. To address this limitation, well-defined crystalline analogues offer a promising route. Wollastonite (CaSiO_3_), which contains chains of corner-sharing SiO_4_ tetrahedra analogous to the *dreierketten* in C–S–H (see Fig. 1), is a structurally simple mineral platform for atomically resolved studies. Theoretical studies on wollastonite have addressed metal–proton exchange^20^ and adsorption on low-energy surfaces.^21–23^ Experimental insights have been limited to spectroscopic investigations of powders or polished aggregates,^24,25^ primarily focusing on the (001) facet. Atomic-scale studies on water adsorption on well-defined surfaces, specifically the lowest-energy (100) surface, have been lacking.

Non-contact atomic force microscopy (nc-AFM) is a unique tool for filling the gaps in the experimental data. The development of qPlus sensors with piconewton force sensitivity^26^ and of functionalized tips enabling sub-molecular resolution^27,28^ provides the basis for the direct visualization of water adsorption at solid surfaces, including insulating materials.^29^ Examples include water dimers, trimers, and tetramers on NaCl(001) thin films supported on Au(111) substrates,^30^ 15-mer clusters on Pt(111) and Cu(111),^31^ 2D bilayer hexagonal ice on Au(111),^32^ water monolayers comprising 5–7-membered rings on Ni(111),^33^ and 1D chains on Cu(110).^34^ Combining precise water dosing in UHV with high-sensitivity force microscopy enables coverage-dependent measurements, which can directly probe computational predictions.

Such a combined approach is crucial, as modeling water structures at surfaces remains challenging. Density functional theory (DFT) predictions are highly sensitive to the choice of exchange-correlation functional. Standard generalized gradient approximation (GGA) functionals systematically underestimate van der Waals dispersion forces, which are crucial for water–surface binding.^35–38^ In contrast, the metaGGA r^2^SCAN functional, combined with the nonlocal rVV10 correlation,^39^ provides improved descriptions that accurately capture both long-range physisorption and short-range interactions.^40^

In this study, the experimental and theoretical challenges are addressed by combining nc-AFM using a qPlus sensor in UHV with DFT calculations employing the metaGGA r^2^SCAN + rVV10 functional. The investigation focuses on water adsorption on the most abundant and environmentally relevant termination of wollastonite, *i.e.*, (100). Different adsorbate structures are resolved, characterized by (1 × 1), (√2 × √2)*R*45°, and (2 × 1) periodicities, and interpreted by DFT with support from AFM simulations.^41^ The competition between water–water and water–surface interactions is crucial to the formation of the adsorption structures.

## Results

This section describes how water structures evolve on wollastonite (100) with increasing coverage: from isolated, strongly bound molecules that follow the lattice symmetry, to interconnected H-bonded networks with distinct symmetries, and finally to water clusters.

### 1 H_2_O per u.c.: nested water

Fig. 1 illustrates the structure and appearance of the wollastonite (100) surface, as determined previously.^42^ The crystal structure consists of chains of corner-sharing SiO_4_ tetrahedra running along the [010] direction, separated by interchain Ca ions. These chains exhibit the *dreierketten* configuration, the fundamental structural building block of C–S–H phases:^5^ a corrugated repeating unit of three corner-sharing tetrahedra. Within this three-tetrahedra period, two (lower) paired tetrahedra point in one direction and a single bridging tetrahedron points in the opposite direction (upper tetrahedron). Upon cleaving, the surface exposes Ca ions arranged in a roughly rectangular unit cell (7.0 Å × 7.3 Å, see Fig. 1a). In nc-AFM images acquired with Cu-terminated tips, the Ca ions appear as dark features, as confirmed by the simulated AFM images based on the DFT model (Fig. 1b). Notably, the UHV-cleaved surface is already hydrated.^42^ Water molecules released from the mineral sample during cleavage at room temperature readily adsorb in the valleys of the surface with a calculated adsorption energy of −1.3 eV per H_2_O, resulting in a coverage of one H_2_O per unit cell. Each of these molecules (henceforth referred to as “nested”) coordinates to two surface Ca ions *via* Ca–O bonds (≈2.53 Å) and donates an H-bond to a bridging oxygen linking the two lower SiO_4_ tetrahedra of the *dreierketten*. While constant-height nc-AFM cannot identify the nested H_2_O molecules because they lie below the most protruding species, their existence is corroborated by the adsorption behavior of CO_2_ investigated in a previous publication, which leads to the formation of surface carbonates. To form the observed carbonates, CO_2_ must bind to the protruding H atoms of the nested water molecules. Without the nested water molecules, CO_2_ remains weakly bound and unreactive, and the experimental data cannot be reproduced.^42^ Interestingly, molecular hydration is energetically favored over dissociative adsorption. In DFT calculations, dissociated water species spontaneously recombined to form intact molecules (Fig. S6a and b), and the relaxation of various hydroxylated surface models resulted in energetically unfavorable configurations (Fig. S6c).

The cleaved surface naturally exhibits defects of currently unknown nature (Fig. 1c), which occupy approximately 2% of the lattice sites; this study focuses on defect-free regions.

### 2 H_2_O per u.c.: protruding water

**Fig. 2:**
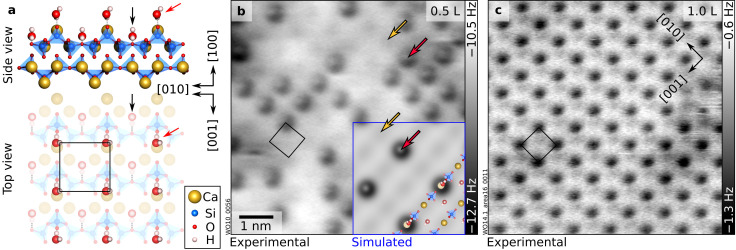
Two H_2_O molecules per unit cell: “protruding” H_2_O. (a) DFT-optimized surface structure of wollastonite (100) with one H_2_O in addition to the “nested” water, for a total of 2 H_2_O molecules per (1 × 1) unit cell. “Nested” and “protruding” water molecules are marked by black and red arrows, respectively. (b and c) 6.9 × 6.9 nm^2^ nc-AFM images corresponding to coverages of “protruding” H_2_O species of ≈ 0.45 ML and 1 ML, respectively. Doses in Langmuir (L) of H_2_O dosed in addition to the nested water are indicated in the upper-right corners of the panels. AFM parameters: (b) *A* = 200 pm, *V*_s_ = −10 V; (c) *A* = 700 pm, *V*_s_ = −3.3 V; Cu-terminated tips. In panel (b), Ca atoms of the wollastonite lattice are faintly visible (yellow arrows) between the protruding water species (red arrows). The overlay in panel (b) shows an AFM simulation derived from the DFT model on the left-hand side (tip–sample distance of 4.5 Å). The AFM simulation was performed on a (2 × 2) supercell derived from the model in panel (a), with 0.25 ML of protruding water molecules per (1 × 1) unit cell (1.25 ML including nested H_2_O). Black rectangles indicate the unit cell.

Previous theoretical studies^21,22^ have also predicted that water adsorption on wollastonite (100) is molecular, albeit with one molecule per unit cell adsorbing atop a Ca ion (with an adsorption energy of −0.9 eV per H_2_O as determined by empirical force-fields employed in ref. 22). As noted above, the first H_2_O molecule per unit cell should instead adsorb in the “nested” configuration because of its stronger adsorption (Fig. 1). Upon placing further molecules on this hydrated surface (for a total of two H_2_O per (1 × 1) unit cell), the configuration predicted by Kundu *et al.* is found (Fig. 2a): H_2_O adsorbs atop Ca ions with one H atom forming an H-bond with a neighbouring O atom of the SiO_4_ tetrahedron, and an adsorption energy of −0.97 eV per H_2_O. The experimental data (Fig. 2b and c) confirm this assignment: upon dosing water at 100 K, additional features become discernible in nc-AFM. Their coverage increases with the dose (Fig. S1), confirming their assignment to water species. At a coverage of roughly 0.45 ML (Fig. 2b), both the new protruding features assigned to water and the Ca ions of the cleaved surface are visible, confirming that protruding H_2_O and Ca ions are in registry. Furthermore, the simulated images based on the theoretical model closely match the experiments (inset in Fig. 2b; see Fig. S1 for a series of images as a function of tip–sample distance). The contrast is dominated by the H atom not engaged in bonding to the surface. Note that the slight offset of the brightest spot in the experimental data compared to the simulations is likely due to tip asymmetry. The distance-dependent agreement (Fig. S1a–c) confirms the proposed configuration.

### 2.0–2.5 H_2_O per u.c.: stripes and stable √2 patches

**Fig. 3:**
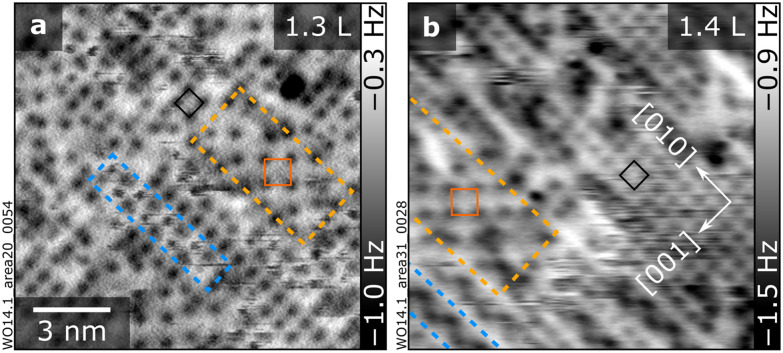
Between 2 and 2.5 H_2_O molecules per unit cell: “stripes” and “stable √2” patches. (a and b) 13.5 × 13.5 nm^2^ nc-AFM images taken with differently terminated tips. Areas with “stripes” and “stable √2” patches are outlined in blue and orange, respectively, and (1 × 1) and (√2 × √2)*R*45° unit cells are marked in black and dark orange, respectively. Doses in Langmuir (L) are indicated in the upper-right corner of the corresponding panels. AFM parameters: (a) *A* = 700 pm, *V*_s_ = −10 V, O-terminated tip; (b) *A* = 700 pm, *V*_s_ = −3.5 V, Cu-terminated tip.

Beyond two molecules per unit cell (*i.e.*, for coverages beyond the completion of a layer of protruding H_2_O with (1 × 1) periodicity as in Fig. 2c), two new patterns emerge (Fig. 3). Both have a local (√2 × √2)*R*45° symmetry and coexist with (1 × 1) patches of protruding H_2_O. The blue and orange dashed rectangles in Fig. 3 highlight these new configurations, henceforth referred to as “stripes” and “stable √2” patches. Their contrast is affected by the tip termination (see Fig. 3a and b, acquired with O- and Cu-terminated tips, respectively, plus additional data in Fig. S2 and S3, which also show their relative alignment compared to the (1 × 1) protruding H_2_O). Despite the same local periodicity, stripes and patches have distinct appearances in nc-AFM.

The stripes appear as isolated, meandering zigzag structures, often ending at defects (see Fig. S3d for a large-scale image with multiple stripes). They run along the [010] direction and are two (1 × 1) unit cells wide along the [001] direction. They tend to interact with the tip and cause the fuzzy appearance seen in Fig. 3. Sometimes the stripes move between consecutively taken images (Fig. S2), suggesting a relatively weak binding of species within the structure.

**Fig. 4:**
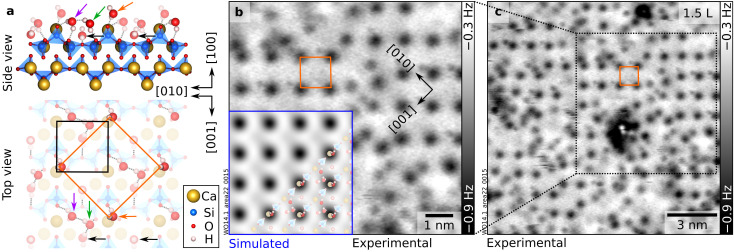
2.5 H_2_O molecules per unit cell: full layer of “stable √2”. (a) DFT-optimized surface structure of wollastonite (100) with a (√2 × √2)*R*45° overlayer containing 2.5 H_2_O per (1 × 1) unit cell (see Fig. S7a for a larger-scale top view of the model). The H-bonded network (marked by gray dotted lines) involves three H_2_O molecules: the nested one (black arrow), one previously protruding (purple arrow), and one additional H_2_O (green arrow). The remaining protruding molecules (orange arrow) are aligned with the surface Ca ions and dominate the AFM contrast (circular dark features). (b and c) nc-AFM images (8.4 × 8.4 nm^2^ and 14 × 14 nm^2^, respectively) corresponding to the same dose of 1.5 L (*A* = 700 pm, *V*_s_ = −2.5 V, O-terminated tip). The inset in panel (b) shows an AFM simulation derived from the DFT model on the left-hand side (tip–sample distance of 5.2 Å).

The “stable √2” patches are also elongated along [010] but are wider than the stripes along [001]. In contrast to the stripes, they are unperturbed by the tip. At low doses, the stripes predominate, and only a few small stable √2 patches are visible with some tips (Fig. S3d). As the coverage increases, the stripes become denser, although they stay mostly separated by one row of (1 × 1) protruding H_2_O features along [010] (*cf.* Fig. S3a and b). At the same time, larger stable √2 patches develop. Eventually, the stable √2 patches dominate the surface, until full coverage is achieved (Fig. 4). A statistical evaluation of nc-AFM images *versus* water doses reveals the same density of H_2_O per (1 × 1) unit cell in the two structures, namely 0.5 extra H_2_O per unit cell compared to the protruding H_2_O of Fig. 2c (corresponding to a total of 2.5 H_2_O, including the nested H_2_O). Although the coverage is now higher, in nc-AFM images, both structures display half the number of features compared to the full layer of protruding H_2_O. As argued below, this is likely caused by the incorporation of the original 2 ML and additional water species into lower-lying configurations that are invisible to constant-height nc-AFM.

Fig. 4a shows a model for the stable √2 discussed in the next section. Using a (√2 × √2)*R*45° simulation cell, a satisfactory structural model for the stripes could not be identified. Under the hypothesis that the stripes might be a metastable structure, an annealing experiment (20 minutes between 120 K and 140 K) was performed on a surface with coexisting stripes and stable √2 patches. No significant changes were observed in the appearances and relative coverages of the structures. It is therefore likely that understanding the stripe structure would require a larger simulation cell, including the rows adjacent to the stripe. However, due to the significant computational effort required to probe many configurations in such a large cell, this approach was not pursued.

### 2.5 H_2_O per u.c.: complete layer of “stable √2”

At a total coverage of 2.5 molecules per (1 × 1) unit cell, an almost complete “stable √2” layer with antiphase domains is observed (Fig. 4b; domain boundaries are highlighted in Fig. S5). As noted above, the density of species detected by nc-AFM is smaller compared to the “protruding H_2_O”, despite the larger H_2_O doses needed to achieve this structure, hence suggesting the presence of lower-lying species that are invisible to nc-AFM in constant-height mode. To establish a structural model of the stable √2 pattern through DFT, 500 candidates were screened (see Computational methods). Several energetically competing models were found, within an energy window of 0.1 eV per unit cell (see Fig. S7 for exemplary configurations and corresponding energies). Such energy differences are too small to be significant, and the inaccessibility of lower-lying species in constant-height nc-AFM prevents a conclusive experimental validation. Nonetheless, most models show common features, with only minor deviations in the orientation of some H atoms (see Fig. S7). Fig. 4a illustrates the lowest-energy structure found. It suggests a cooperative reorganization of the hydration layer. The extra H_2_O added to the “protruding” configuration induces a structural reorganization: every second protruding H_2_O moves to a lower position where it accepts one H-bond from the new H_2_O and donates one to the apical O atom of the surface SiO_4_ tetrahedron (purple arrow). At the same time, the new H_2_O (green arrow) accepts an H-bond from the nested H_2_O. The presence of multiple, lower-lying species explains the lower number of species observed in nc-AFM: the contrast is dominated by the remaining protruding H_2_O (Fig. 4b). While it is impossible to confirm the arrangement of low-lying species, AFM image simulations based on this proposed model are consistent with experiment.

### 2.5–4 H_2_O per u.c.: (2 × 1)

**Fig. 5:**
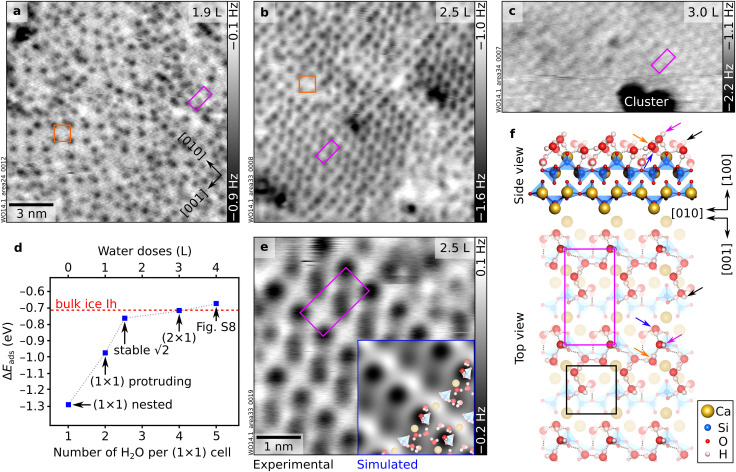
Between 2.5 and 4 H_2_O molecules per unit cell: from “stable √2” to (2 × 1) configurations. (a and b) 15 × 15 nm^2^ nc-AFM images of wollastonite (100) with increasing H_2_O coverages, moving from (a) the “stable √2” (orange square) plus additional features to (b) a (2 × 1) overlayer. Water doses in Langmuir (in addition to the nested water) are indicated in the top-right corners of the AFM images. (c) 14.7 × 7.6 nm^2^ nc-AFM image showing a water cluster (dark; attractive interaction with the tip) on top of the (2 × 1) layer. (d) Differential adsorption energies (Δ*E*_ads_) of the calculated configurations at different H_2_O coverages. The dashed red line indicates the bonding energy of hexagonal bulk ice calculated with the same functional; the dotted, grey line connects the blue dots to visualize the trend. The blue markers correspond to the structure models of “nested” H_2_O (Fig. 1a), “protruding” H_2_O (Fig. 2a), full “stable √2” pattern (Fig. 4a), (2 × 1) pattern (panel f), and the lowest-energy model obtained for a coverage of five H_2_O per (1 × 1) unit cell (Fig. S8). (e) 5 × 5 nm^2^ nc-AFM image of the (2 × 1) structure overlaid by the AFM simulation corresponding to the model in panel (f). AFM parameters: (a) *A* = 700 pm; *V*_s_ = −3.6 V; (b) *A* = 700 pm; *V*_s_ = −0.1 V; (c) *A* = 700 pm; *V*_s_ = −3.4 V (O-terminated tips). Orange squares and magenta rectangles identify the unit cells of the “stable √2” and (2 × 1) structures, respectively. (f) DFT-optimized structure model of wollastonite (100) with 4 H_2_O per (1 × 1) unit cell, forming a (2 × 1) pattern (see Fig. S7d for a larger-scale top view of the model). Magenta and black arrows indicate the most and second-most protruding H_2_O molecules, respectively. Blue and orange arrows mark the two neighboring molecules donating H bonds to the most protruding H_2_O.

Upon dosing further water on the complete “stable √2” layer, a new configuration with (2 × 1) periodicity emerges (see Fig. 5 and Fig. S5 for the transition between the two structures). The new (2 × 1) structure contains four H_2_O per (1 × 1) unit cell, as suggested by the experimental doses. It is characterized by [010]-oriented rows containing one dark, circular feature and one fainter, elongated feature per (2 × 1) unit cell (see the close-up image in Fig. 5e). Similar to the “stable √2” modeling, several of the candidates screened in DFT were found to be structurally and energetically close (Fig. S7). Fig. 5f shows the lowest-energy model. The most protruding H_2_O (magenta arrow) donates an H-bond to the apical O atom of an upper surface SiO_4_ tetrahedron while accepting H-bonds from two neighboring molecules. The first of these neighbors (blue arrow) accepts an H-bond from a nested H_2_O and donates an H-bond to another O of the upper SiO_4_ tetrahedron. The second neighbor (orange arrow) is involved in an extended H-bonding network that involves lower Si tetrahedra of the *dreierketten* and includes the second-most protruding H_2_O species. This species accepts a bond from a nested H_2_O and donates to an apical O of the upper tetrahedron of the next row of *dreierketten*. Notably, in all low-energy structures, the nested H_2_O molecules remain rigidly bound to their original sites, underscoring their exceptional stability. This arrangement rationalizes the experimental contrast with (2 × 1) periodicity (see Fig. 5e, overlaid to the AFM simulation of the proposed model): the most protruding H_2_O molecule in each (2 × 1) unit cell (magenta arrow) appears as a circular, prominent dark feature, while the molecule marked by a black arrow is seen as a fainter, elongated feature. Water dosed to the (2 × 1) structure does not trigger the formation of any new surface structure. Instead, three-dimensional clusters form on top of the (2 × 1)-ordered surface (see Fig. 5c).

## Discussion

The literature on water–oxide interactions highlights their inherent complexity, as varied cation coordination on oxide surfaces, structural defects, and local electrostatic fields result in a broad spectrum of adsorption structures. For instance, water on rutile TiO_2_(110) forms H-bonded dimers on hydroxylated surfaces;^10^ MgO(001) develops ordered monolayer structures with partial dissociation;^11^ CaO(001) exhibits partial dissociation and hydroxylation, forming ordered chains and networks at room temperature.^12,13^ Half-dissociated monolayers coexist with molecular configurations on ZnO(101̅0).^14^ At low coverages, In_2_O_3_(111) develops ordered hydroxyl overlayers,^15^ while on Fe_3_O_4_(001), partially dissociated dimers act as anchors for extended H-bonded networks.^16^ The intricacy demonstrated by these studies is amplified in more complex minerals. On calcite (CaCO_3_), water preferentially binds to carbonate group rows on the (2 × 1)-reconstructed surface, forming islands between 0.5 and 1 ML coverage that integrate into a (1 × 1) monolayer structure at full coverage.^43^ Some silicate minerals, such as microcline feldspar (KAlSi_3_O_8_), undergo spontaneous hydroxylation at room temperature, in turn facilitating ordered water adsorption.^44^ The formation of specific patterns on each system is governed by the complex interplay between water–water and water–surface interactions, which is modulated by the atomic details of the surface structure.

The wollastonite CaSiO_3_(100) surface provides an opportunity to disentangle these contributions experimentally. At the lowest coverages, water molecules adsorb molecularly and occupy well-defined Ca sites, forming (1 × 1) motifs, characterized by “nested” and “protruding” water (Fig. 1 and 2). The unusually large adsorption energies calculated for 1–2 H_2_O per unit cell (−1.3 eV and −0.97 eV, respectively) indicate that electrostatic interactions with surface Ca^2+^ dominate in this regime. The nested water molecules maximize direct contact with the cations and act as anchors for adsorption at intermediate coverages, in close analogy to hydroxyl groups serving as nucleation points for H-bonded networks on other oxides.^15^ Importantly, no evidence for water dissociation is found at any coverage, in agreement with earlier theoretical predictions^21,22^ but distinct from the behavior of the (001) termination.^23–25^ As recently demonstrated by direct atomic-scale measurements on model oxides, different surface oxygen sites can exhibit distinct proton affinities, which govern the surface's tendency to accept or abstract a proton from an adsorbate.^45^ On the wollastonite (100) surface, the relatively low proton affinity (*i.e.*, limited Brønsted basicity) of the surface oxygen atoms disfavors proton transfer from water and thus suppresses dissociative adsorption.

Above 2 ML (nested and protruding water adsorbed), more complex interactions and patterns emerge. The new molecules do not simply decorate every equivalent site, like at lower coverages where water–surface interactions dominate. The added H_2_O interacts with the nested H_2_O such that translational symmetry of the lattice is broken. Water–water interactions become more important in the new hydration patterns (“stable √2” and “stripes”, see Fig. 3 and 4), ultimately giving rise to denser (2 × 1) arrangements and, beyond ≈4 H_2_O per unit cell, to three-dimensional clusters (Fig. 5c).

The differential adsorption energy for the addition of the fifth H_2_O molecule per unit cell (see Fig. S8) is −0.68 eV, which is less stable than the cohesive energy of bulk ice Ih calculated with the same method (−0.71 eV; see Fig. 5d). This energetic crossover demonstrates that the system has shifted from a surface-templated regime into one governed by water–water cohesion, providing a thermodynamic rationale for the emergence of three-dimensional clusters. The onset of three-dimensional growth rather than the formation of extended ice-like overlayers can be understood by considering both water density and lattice symmetry. At the cluster-nucleation threshold, the surface water density is well below that of the ice Ih basal plane (see Table S2). Moreover, the wollastonite (100) surface imposes a rectangular lattice constraint that is incompatible with the hexagonal symmetry of bulk ice Ih, the thermodynamically stable crystalline phase at the investigated low-pressure conditions. This fundamental mismatch hinders epitaxial ice growth, in contrast, *e.g.*, to the facile epitaxy supported by the AgI(0001) surface.^46^

The complexity of water adsorption on wollastonite (100) is underscored by the multivalley energetic landscape revealed by DFT: a multitude of distinct, competing configurations exist at coverages beyond two H_2_O per u.c. (Fig. S7). These structures lie within a narrow energy window (<0.1 eV per u.c.) that falls within the typical accuracy limits of DFT for H-bonded systems.^38^ This energetic near-degeneracy makes it challenging to pinpoint a unique ground-state structure. Instead, it points toward a family of structurally similar and energetically close configurations. This theoretical finding mirrors the coexisting structural motifs observed experimentally as well as the absence of sharp phase transitions.

Although standard ground-state DFT calculations are inherently limited in that they map the potential-energy surface at 0 K, they remain an indispensable tool for broad structural screening. Alternatives such as *ab initio* molecular dynamics (AIMD) could incorporate finite-temperature effects, but their computational cost precluded their use for the extensive structural screening required by the system investigated here. Machine-learned force fields (MLFFs) could offer an alternative thanks to the accessible timescales for dynamic simulations; however, when applied to multicomponent systems such as the one in the present investigation, they still suffer from limitations concerning absolute energy accuracy and long-range interactions.^47,48^ The main limitation of standard ground-state DFT is that it often yields multiple near-degenerate configurations separated by only a few meV.^49^ In such multi-valley landscapes, entropic contributions and kinetic barriers can stabilize slightly higher-energy configurations observed in finite-temperature experiments.^16^ Pairing 0 K DFT predictions with structural symmetry constraints from nc-AFM helps mitigate these limitations.

Also note that this study focuses on defect-free regions of a model silicate mineral. Real mineral surfaces and industrial cements are inherently more complex and heterogeneous, featuring, *e.g.*, vacancies and substitutional defects that are expected to promote hydroxylation and hydration. Nevertheless, the interaction of the pristine (100) terrace of wollastonite with water investigated here establishes a benchmark for understanding how water interacts with more complex silicate systems.

## Conclusions

This study reveals the structural evolution of water adsorbed on a model calcium silicate surface, wollastonite CaSiO_3_(100). A combination of atomically resolved nc-AFM and DFT demonstrates that water adsorbs molecularly with distinct motifs governed by the coverage-dependent competition between water–surface and water–water interactions.

At low coverage (one to two H_2_O per unit cell), the adsorption geometry is strictly governed by water–surface interactions, characterized by molecules anchored *via* electrostatic forces to surface calcium ions and hydrogen bonds to the silicate framework. In the intermediate regime (two to four H_2_O per unit cell), the increased water density triggers a competition between adhesion and intermolecular cohesion, resulting in a flat potential-energy landscape populated by coexisting, complex hydrogen-bonded networks. Finally, at coverages exceeding four molecules per unit cell, water–water cohesion overcomes surface templating effects, leading to the nucleation of three-dimensional clusters.

The results provide mechanistic insight into how water interacts with calcium silicate surfaces, establishing a fundamental framework for understanding processes such as cement hydration and aqueous weathering. The near-degeneracy in energy of the coexisting surface phases poses a challenge for theoretical simulations. In addition, nc-AFM solely probes the uppermost, protruding water molecules rather than the full adsorption geometry. Nevertheless, the symmetry revealed by the experiments constrain the range of viable configurations and guide the computational analysis toward the establishment of structural models. Overall, this work demonstrates a robust symmetry-guided strategy for unraveling complex oxide–water interfaces.

## Methods

### Experimental methods

#### Sample and *ex situ* characterization

A crystalline sample of wollastonite 1A was extracted from a larger specimen obtained from a skarn occurrence in Turkey. The specimen was previously characterized and analyzed by light and polarization microscopy, electron probe microanalysis, in-air AFM, X-ray diffraction, and electron back-scattered diffraction (EBSD).^42^ Throughout this work, we adopted the conventional unit cell notation (bulk lattice parameters should fulfill *a* > *b* > *c*), which is standard in mineralogical databases and other works^21,22,42,50,51^ and leads to the labeling of the lowest-energy surface as (100). The (100)-oriented grains (see EBSD analysis in ref. 42 for the orientation determination) were glued onto Omicron-style stainless-steel plates using a UHV-compatible epoxy glue (EPO-TEK T7110-38), and a metal stud was glued to the top of the sample. A tangential force applied to the stud cleaved off the portion of the sample initially covered by the stud.^44^ During cleaving, the pressure in the UHV chamber spiked by a few orders of magnitude, as previously reported for this and other silicate minerals.^42,44^ An optical microscope attached to the UHV chamber helped identify portions of the cleaved sample that were flat enough to approach the nc-AFM tip.

#### UHV setup

The experiments were carried out in a UHV setup consisting of two interconnected chambers: a preparation chamber for sample cleaving, gas dosing at 100 K, and X-ray photoelectron spectroscopy (XPS) with a base pressure below 1 × 10^−10^ mbar, and an adjacent chamber for nc-AFM (1 × 10^−11^ mbar). Ultrapure water (MilliQ, Millipore, 18.2 MΩ cm, ≤3 ppb total organic carbon) was dosed into the preparation chamber through a leak valve while keeping the sample at 100 K. The water was purified through several freeze–pump–thaw cycles.^52^ The amount of water deposited was estimated based on the dose (partial pressure × time) required to achieve a coverage of one molecule per unit cell (u.c.), as determined by counting features on the surface in the nc-AFM images and assuming a coverage-indipendent sticking probability at 100 K. This method yielded an approximate coverage of 1 H_2_O per unit cell (u.c.) per 1 Langmuir (L) dosed on top of the as-cleaved surface (1 L = 1.33 × 10^−6^ mbar s). Nc-AFM confirmed that all dosed water molecules desorbed from the surface after warming up to room temperature. One monolayer (ML) is defined as one molecule per (1 × 1) unit cell.

#### Nc-AFM

The nc-AFM measurements were performed in constant-height mode at 78 K, using a commercial Omicron qPlus low-temperature (LT) head and a differential cryogenic amplifier.^53^ The qPlus AFM sensors (*k* = 2000–3500 N m^−1^, *f*_0_ ≈ 27 or 32 kHz, *Q* ≈ 14 000 or 12 000) with a separate contact for the tunneling current had etched W wire tips glued to the oscillating prong of the sensor. In the UHV chamber, the tip was prepared in STM mode on a partially oxidized Cu(110) surface.^28^ Soft indentation and voltage pulses were repeated until atomically sharp tips with a frequency shift typically less than −1.5 Hz were achieved at the tunneling settings of 500 mV and 200 pA. The tips were terminated with Cu or O as judged by the contrast in constant-current STM.^42^ This enabled chemically sensitive AFM and comparison to simulated images derived from density functional theory (see Computational methods). Note that the constant-height mode makes AFM sensitive only to the most protruding species.

To mitigate surface charges developed during UHV cleaving, the sample was irradiated for one minute with X-rays from the XPS setup.^44^ Residual charges were assessed using the Kelvin parabola method^54^ and compensated by applying a bias voltage, *V*_s_, to the back of the sample plate while keeping the tip potential close to ground.

#### Data analysis

Raw images were processed in ImageJ^55^ to correct distortions arising from piezo creep and thermal drift^56^ and to remove low-frequency noise. Limited sampling areas, tip-dependent imaging contrast (Fig. 3 and S4), and uncertainties in the dosing procedure may introduce errors in the estimated water densities for each structure. Nevertheless, the estimates are considered reliable, as experiments performed with incremental dosing yielded results consistent with those obtained using a single, larger dose.

### Computational methods

#### Calculation details

DFT calculations were performed using the projector-augmented wave (PAW) method,^57,58^ as implemented in the Vienna *Ab initio* Simulation Package (VASP, version 6.5.1).^59^ The metaGGA r^2^SCAN + rVV10^36,37,39^ exchange-correlation functional was employed, unless otherwise noted. These functionals were chosen for their good description of the bulk properties of wollastonite, with lattice parameters and angles deviating by less than 0.5% from the values measured by XRD (see Table S1). The bulk structure was optimized using a cutoff energy of 800 eV, a 3 × 3 × 3 *k*-point mesh for Brillouin zone integration, and a Gaussian smearing with a width of 0.1 eV. This higher cutoff energy was chosen to mitigate Pulay stress^60^ during variable-cell relaxation. For the (100) surface calculations, symmetric slabs were constructed. They had a thickness of three bulk unit cells (*i.e.*, 18 formula units of CaSiO_3_, 90 atoms in total) separated by 15 Å vacuum spacing. These surface calculations employed a lower cutoff energy of 500 eV and a 1 × 3 × 3 *k*-point mesh for (1 × 1) cells. All calculations utilized the default precision setting (PREC = normal). All atoms were free to relax. The geometries were optimized using the conjugate gradient method until the residual forces on the atoms were smaller than 0.01 eV Å^−1^. The criterion for electronic convergence was set to an energy change of less than 10^−6^ eV. Differential adsorption energies (Δ*E*_ads_), defined as the energy required to add Δ*n* molecules to a surface already containing *n* pre-adsorbed molecules, were calculated according to:
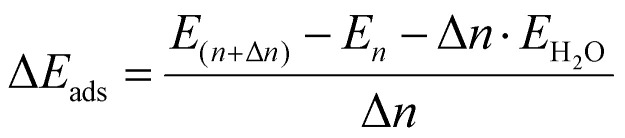
where *E*_(*n*+Δ*n*)_ and *E*_*n*_ were the relaxed total energies of the surface slab after and before the addition of Δ*n* molecules, respectively. *E*_H_2_O_ was the total energy of an isolated water molecule in the gas phase.

#### Structure search strategy

Water adsorption configurations were investigated at coverages between 1 and 5 H_2_O molecules per (1 × 1) unit cell. The dimensions of the computational supercells were selected to replicate the experimentally observed periodicities. Accordingly, (1 × 1) cells were employed to model “nested” (1 H_2_O per u.c.) and “protruding” (2 H_2_O per u.c.) water in the low-coverage regime. The “stable √2” (2.5 H_2_O per u.c.) and (2 × 1) (4 H_2_O per u.c.) patterns were simulated using (√2 × √2)*R*45° and (2 × 1) unit cells, respectively. Throughout this work, surface patterns are described using the standard surface science convention, which lists the scaling factor for the shorter surface lattice vector first. Consistent with the loss of long-range order in nc-AFM images at exposures exceeding 3 L (>4 H_2_O per u.c.), a standard (1 × 1) unit cell was adopted to investigate the high-coverage limit of 5 H_2_O molecules. To reduce computational cost for the structure search, the wollastonite substrate was modeled using a symmetric slab with a thickness of one unit bulk cell (*i.e.*, 6 CaSiO_3_ formula units, 30 atoms in total per (1 × 1) unit cell), thinner than the one used for obtaining the final optimized structure models and the calculated adsorption energies. The atoms in the lower half of this slab were frozen in the bulk positions. Before studying adsorption, an individual water molecule was optimized in the gas phase within an 8 Å cubic unit cell. Subsequently, the optimized molecules were placed in randomized starting positions to avoid a systematic bias by randomizing both the center of mass and the 3D orientation of each molecule *via* a rotation matrix. These placements were constrained such that all atoms of the generated water molecules were located at a height between 1.5 Å and 4.5 Å above the wollastonite surface. To avoid non-physical structures, which would cause large repulsive forces during the DFT relaxations, the minimum distance between all atoms (intermolecularly and between water molecules and the surface) was set to 1.5 Å. A two-stage relaxation strategy was employed to efficiently screen the potential-energy surface and reach more favorable configurations. For a preliminary screening, approximately 500 randomly generated structures per water coverage were pre-relaxed using the GGA PBE-D3 exchange-correlation functional.^61^ This functional was selected because it is computationally less demanding than metaGGA functionals, explicitly includes van der Waals interactions, and is a good compromise for modeling solid–water interfaces.^46^ Following this preliminary screening, the lowest-energy candidate structures were selected for final and accurate relaxation using the more expensive metaGGA r^2^SCAN + rVV10 functional and symmetric slabs with a thickness of three bulk unit cells (see the DFT-optimized structure files St1–St5 included in the SI). Consistency checks were performed on some configurations to ensure the reliability of the PBE-D3 functional for the pre-relaxation step. All final adsorption energies and optimized structures were calculated at the r^2^SCAN + rVV10 level of theory.

#### AFM simulations

Non-contact AFM images of the relaxed models were simulated using the Probe-Particle Model,^41,62,63^ which includes the electrostatic potential above the surface (derived from the DFT calculation), Lennard-Jones potentials, and the elastic properties of the tip. Cu, CuOx, and CO tips were simulated using the following values of lateral and vertical spring constants and charges (Cu: *k*_*x*,*y*_ = 0.75 N m^−1^, *k*_*z*_ = 50.7 N m^−1^, effective tip charge −0.05*e*; CuOx: *k*_*x*,*y*_ = 161.9 N m^−1^, *k*_*z*_ = 271.1 N m^−1^, effective tip charge −0.05*e*; CO: *k*_*x*,*y*_ = 1.7 N m^−1^, *k*_*z*_ = 326.9 N m^−1^, effective tip charge −0.005*e*). The experimental oscillation amplitude was used in the simulations. Since the exact height of the tip in the experiment is unknown, a height was chosen that yielded the best visual agreement between the experiment and the simulation. Importantly, while varying this height modulates the overall AFM contrast intensity, the simulated spatial symmetry and lateral feature positions are dictated by the DFT-derived electrostatic potential, ensuring reliable structural assignments.

## Author contributions

Conceptualization: GF. Data curation: AC, GF, LL. Formal analysis: AC, GF, LL. Funding acquisition: GF, UD. Investigation: AC, LL, GF, AH, EV, DAA. Project administration: GF, UD. Resources: RA. Software: AC, LL, GF, FM, MS. Supervision: UD, GF, FM. Validation: RA, UD, GF, MS, FM. Visualization: AC, LL, GF. Writing – original draft: AC, GF, LL. Writing – review & editing: all authors.

## Conflicts of interest

There are no conflicts to declare.

## Supplementary Material

NR-018-D6NR01063F-s001

## Data Availability

The data supporting this article have been included as part of the supplementary information (SI). Supplementary information: additional nc-AFM data, AFM simulations, and computational results, plus structure files. See DOI: https://doi.org/10.1039/d6nr01063f.
