# Supplementary material for: Water adsorption on a model silicate surface: wollastonite (100)
Source: Nanoscale. 2026 May 16;18(25):13422–32. doi: 10.1039/d6nr01063f (PMC13217135; doi:10.1039/d6nr01063f)
Supplement: NR-018-D6NR01063F-s001 [file NR-018-D6NR01063F-s001.pdf]

Supplementary Information (SI) for Nanoscale.
This journal is © The Royal Society of Chemistry 2026

Supporting Information for

Water adsorption on a model silicate surface: wollastonite (100)

Luca Lezuo,[1†] Andrea Conti,[1†] Alexander Hoheneder,[1] Elena Vaníčková,[2] Domitilla Alessandra Aloi,[1] Rainer Abart,[3] Florian Mittendorfer,[1] Michael Schmid,[1] Ulrike Diebold,[1] Giada Franceschi[1*]

[1]Institute of Applied Physics, TU Wien, 1040, Vienna, Austria
[2]Central European Institute of Technology, Brno University of Technology, 61200, Brno, Czech Republic
[3]Department of Lithospheric Research, Universität Wien, 1090, Vienna, Austria
[†] These authors contributed equally.

*Corresponding author: Giada Franceschi
Email: franceschi@iap.tuwien.ac.at

This PDF file includes:

Section S1: Additional nc-AFM data and AFM simulations
- Figure S1: Protruding water molecules at different tip-sample distances and coverages
- Figure S2: Mobility of water species within the "stripes"
- Figure S3: Increasing density of stripes
- Figure S4: Relative alignment of different water structures
- Figure S5: More details about the transition between the "stable √2" and the (2 × 1) structure

Section S2: Additional computational results
- Figure S6: Energetic preference for molecular water adsorption on the pristine wollastonite surface
- Figure S7: Comparison of energetically similar water configurations for the "stable √2" and (2 × 1) structures
- Figure S8: Lowest-energy model with a coverage of five H_2O per (1 × 1) unit cell
- Table T1: Experimental and calculated bulk properties of wollastonite
- Table T2: Surface water density for bulk ice Ih, silver iodide and wollastonite

Structure files (separate files):

- St1: (1 × 1) nested H_2O model (Fig. 1a). File name: "St1_1x1_nested.cif"
- St2: (1 × 1) protruding H_2O model (Fig. 2a). File name: "St2_1x1_protruding.cif"
- St3: "stable √2" model (Fig. 4a). File name: "St3_stable_sqrt2.cif"
- St4: (2 × 1) model (Fig. 5f). File name: "St4_2x1.cif"
- St5: (1 × 1) model with $5H_2O$/u.c. (Fig. S8). File name: "St5_1x1_5H2O.cif"

Section S1: Additional nc-AFM data and AFM simulations

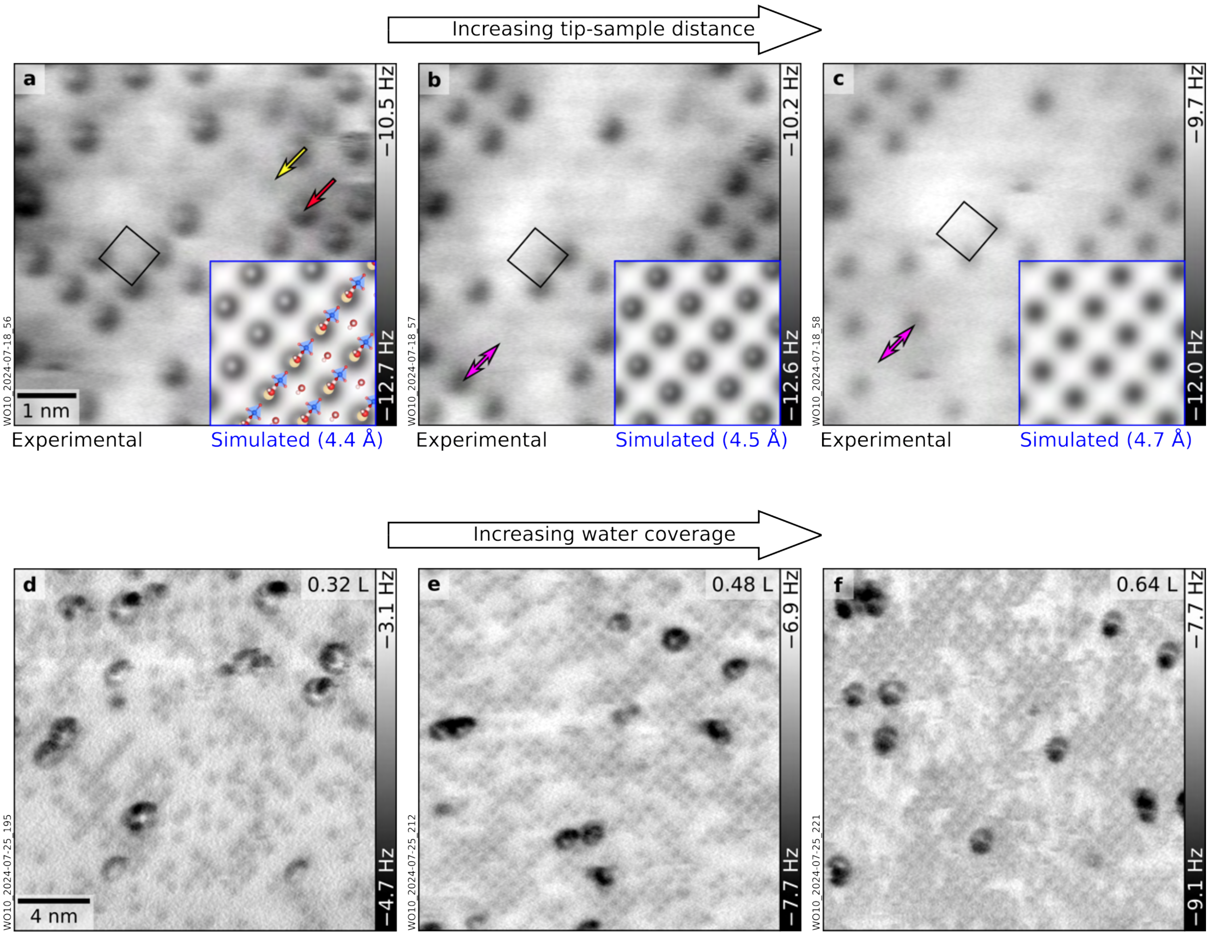

Fig. S1: Protruding water molecules at different tip-sample distances and coverages. (a–c) Consecutive nc-AFM images (6.1 × 6.1 nm^2) taken with increasing tip–sample distance (20 pm between each frame) on a surface partially covered with protruding water molecules. Surface Ca atoms are faintly visible (yellow arrow); the protruding water (red arrow) sits at equivalent lattice positions. Some molecules (magenta double arrows) undergo changes in position between consecutive images. Insets: AFM simulations based on the model shown in Figure 2a of the main text (tip–sample distances in brackets). (d–f) 20.0 × 20.0 nm^2 nc-AFM images of the cleaved surface after water exposure to (d) 0.32 L (resulting in a coverage of 0.33 ML), (e) 0.48 L (0.55 ML), and (f) 0.64 L (0.75 ML). AFM parameters: (a–c) A = 200 pm, V_s = −10 V; (d) A = 540 pm, V_s = −10 V; (e) A = 580 pm, V_s = −10 V; (f) A = 480 pm, V_s = −10 V.

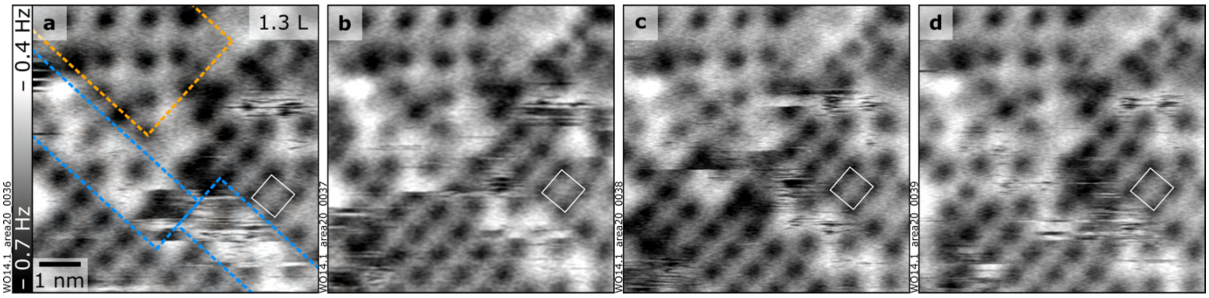

Figure S2: Mobility of water species within the "stripes". (a–d) Consecutive nc-AFM images (7 × 7 nm^2) of wollastonite (100) covered with 2.3 H_2O molecules; the white square marks the (1 × 1)-unit cell in the same spot. $\Delta t \approx 12.5$ min between successive images. (a) Dashed blue rectangles indicate stripes, while the dashed orange rectangle indicates a stable √2 patch. A = 700 pm, V_s = −10.0 V, O-terminated tip.

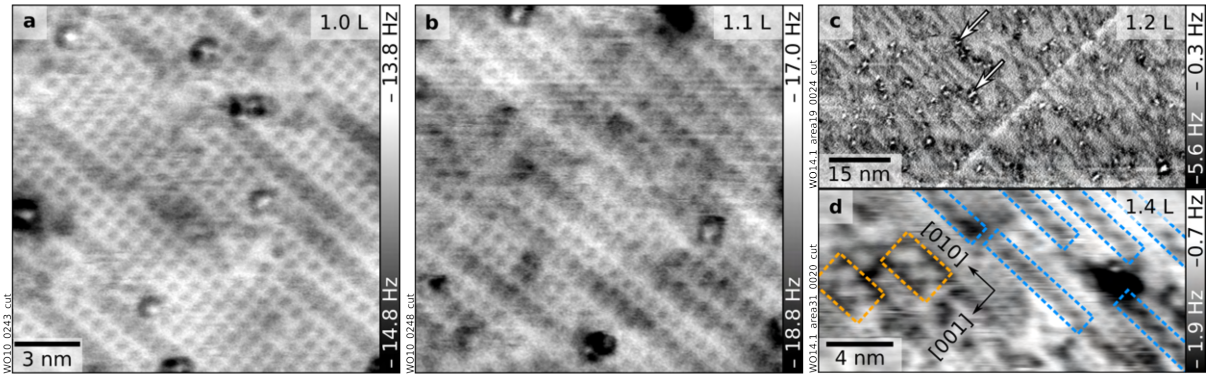

Figure S3: Increasing density of stripes. (a–d) nc-AFM images of wollastonite (100) with increasing coverages of water (doses in Langmuir, in addition to the nested water, are indicated at the top right corner of each panel). (a, b) 17 × 17 nm^2 images showing the increasing density of the stripes with increasing coverage (the stable √2 patches are not resolved with this tip). (c) 90 × 45 nm^2 image: The stripes often end at surface defects (arrows). (d) 22 × 11 nm^2 image highlighting the coexistence of stripes (blue markings) and √2 patches (orange markings), which can be distinguished with this tip. AFM parameters: (a) A = 490 pm, V_s = −10.0 V; (b) A = 400 pm, V_s = –10.0 V; (c) A = 700 pm, V_s = −8.5 V; (d) A = 480 pm, V_s = −3.5 V, Cu-terminated tip.

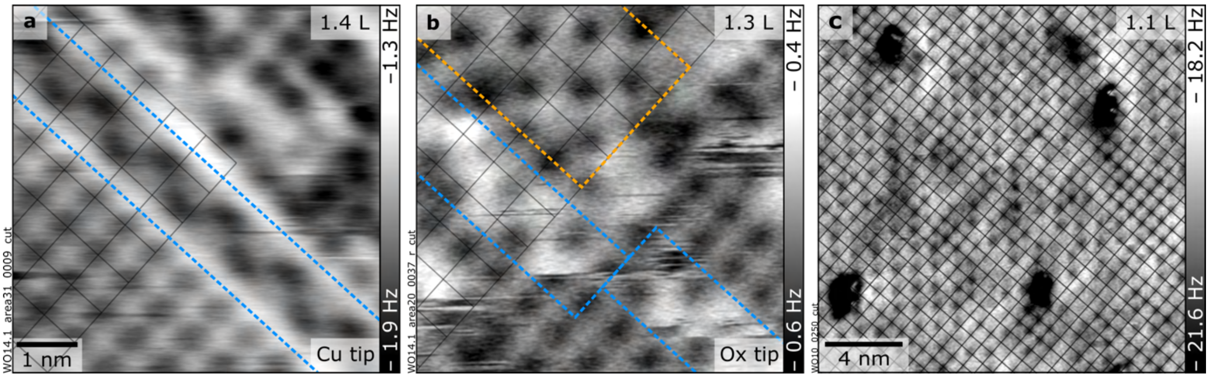

Figure S4: Relative alignment of different water structures. (a, b) 6.5 × 6.5 nm^2 and (c) 19.0 × 19.0 nm^2 nc-AFM images of wollastonite (100) plus water at a coverage of 2.1–2.4 H_2O molecules per (1 × 1) unit cell. Doses in Langmuir, in addition to the nested water, are shown at the top-right corners of the corresponding panels. The dark spots with (√2 × √2)R45° periodicity are shifted with respect to the (1 × 1) unit cell (black grid); note that the magnitude of the apparent shift depends on the tip termination. Stripes and stable √2 patches are highlighted in blue and orange in panels (a) and (b), respectively; the tip in panel (c) does not distinguish between the (1 × 1) areas and the stripes. AFM parameters: (a) A = 700 pm, V_s = −3.5 V, Cu-terminated tip; (b) A = 700 pm, V_s = −10.0 V, O-terminated tip; (c) A = 350 pm, V_s = −10.0 V, wollastonite-modified tip.

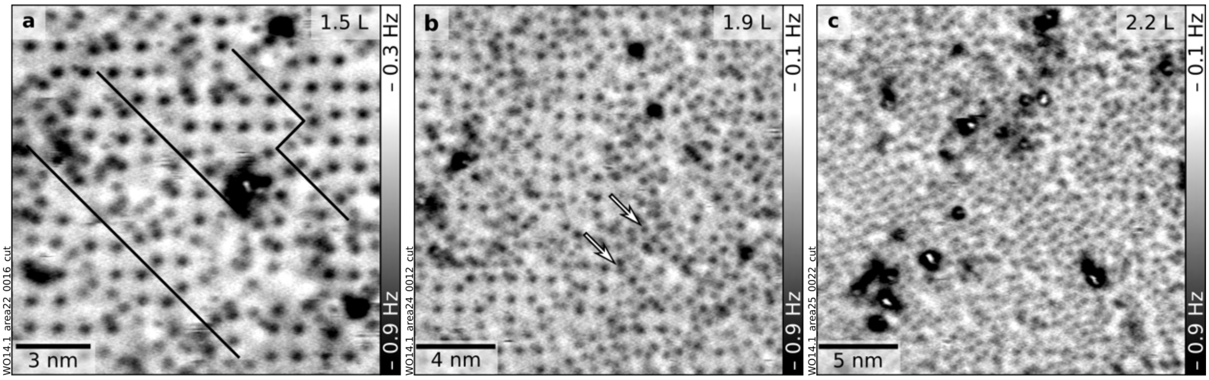

Figure S5: More details about the transition between the "stable √2" and the (2 × 1) structure. (a–c) 15 × 15, 19 × 19, and 23 × 23 nm^2 nc-AFM images of coverages between 2.5 and 3.2 H_2O molecules per (1 × 1) unit cell (doses in Langmuir, in addition to the nested water, are indicated in the top-right corners). (a) Slightly beyond the full "stable √2" pattern: Black lines highlight domain boundaries. (b) A preferential ordering along the [010] direction becomes apparent (see arrows). (c) Nearly complete (2 × 1) layer. AFM parameters: (a) A = 700 pm, V_s = −2.7 V; (b) A = 700 pm, V_s = −3.6 V; (c) A = 700 pm, V_s = −2.5 V; O-terminated tips.

Section S2: Additional computational results

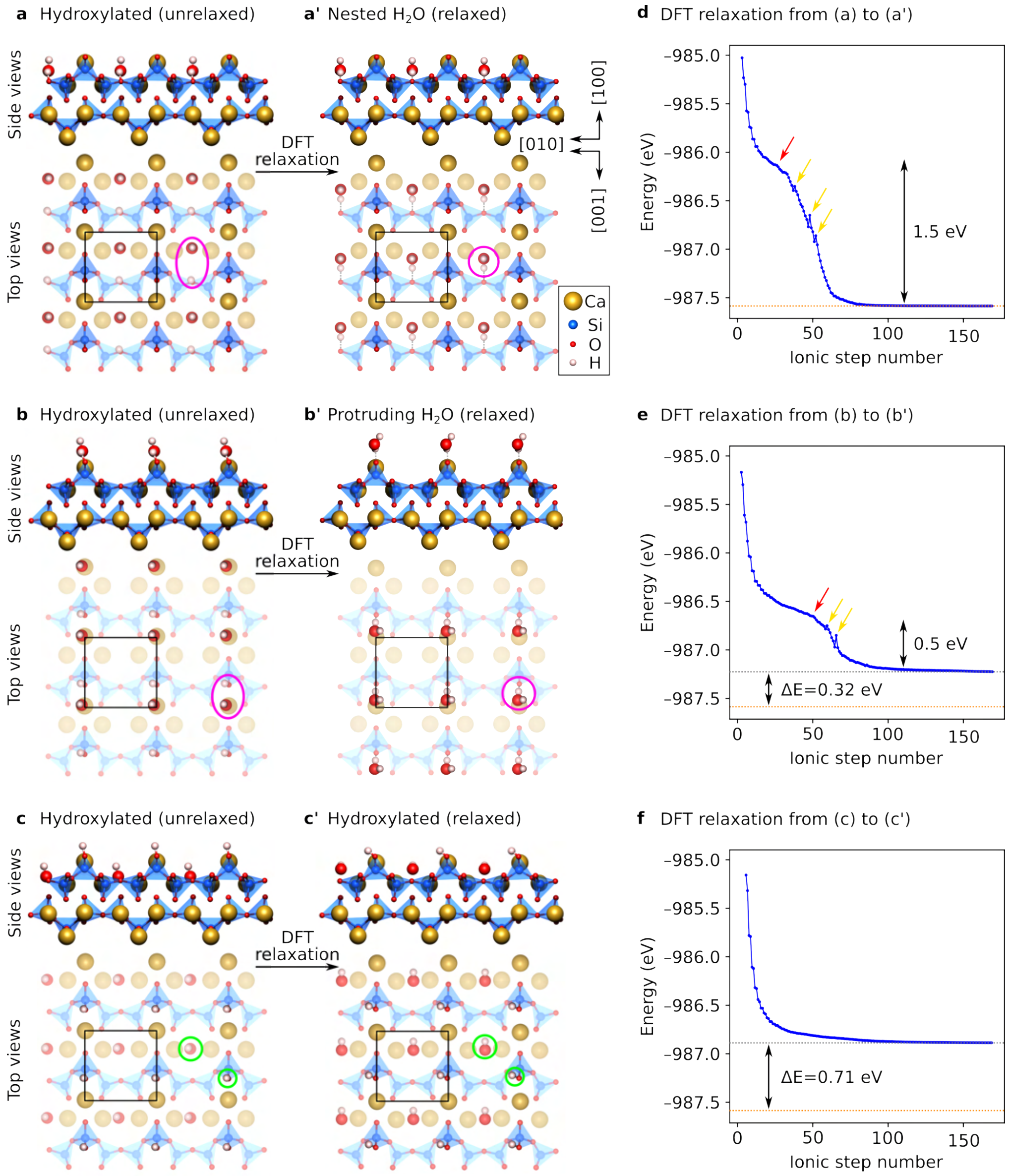

Figure S6: Energetic preference for molecular water adsorption on the pristine wollastonite surface. (a–c) Initial unrelaxed configurations representing hypothetical dissociative adsorption states, with hydroxyl groups and protons coordinated to surface Ca ions and bridging or apical oxygens of silica tetrahedra, respectively. (a'–c') Final relaxed configurations obtained after DFT optimization at 0 K. Dissociated species spontaneously recombine into intact water molecules in (a') nested or (b') protruding configurations (magenta circles); (c') widely separated species remain dissociated (green circles). (d–f) Energy profiles along the relaxation path from the dissociated states (a–c) to the molecular states (a'–b') and hydroxylated state (c'). Transient energy spikes (yellow arrows) reflect conjugate gradient trial steps probing repulsive regions during O–H bond formation. Red arrows indicate the onset of molecular recombination. The nested molecular state is the most stable (dotted orange line), while protruding molecular and fully hydroxylated states are 0.32 eV/H_2O and 0.71 eV/H_2O less favorable, respectively. The significant energy gain upon water recombination (0.5–1.5 eV) demonstrates that molecular adsorption is favored over dissociation, in contrast to other silicate surfaces[1] or wollastonite (001).[2,3]

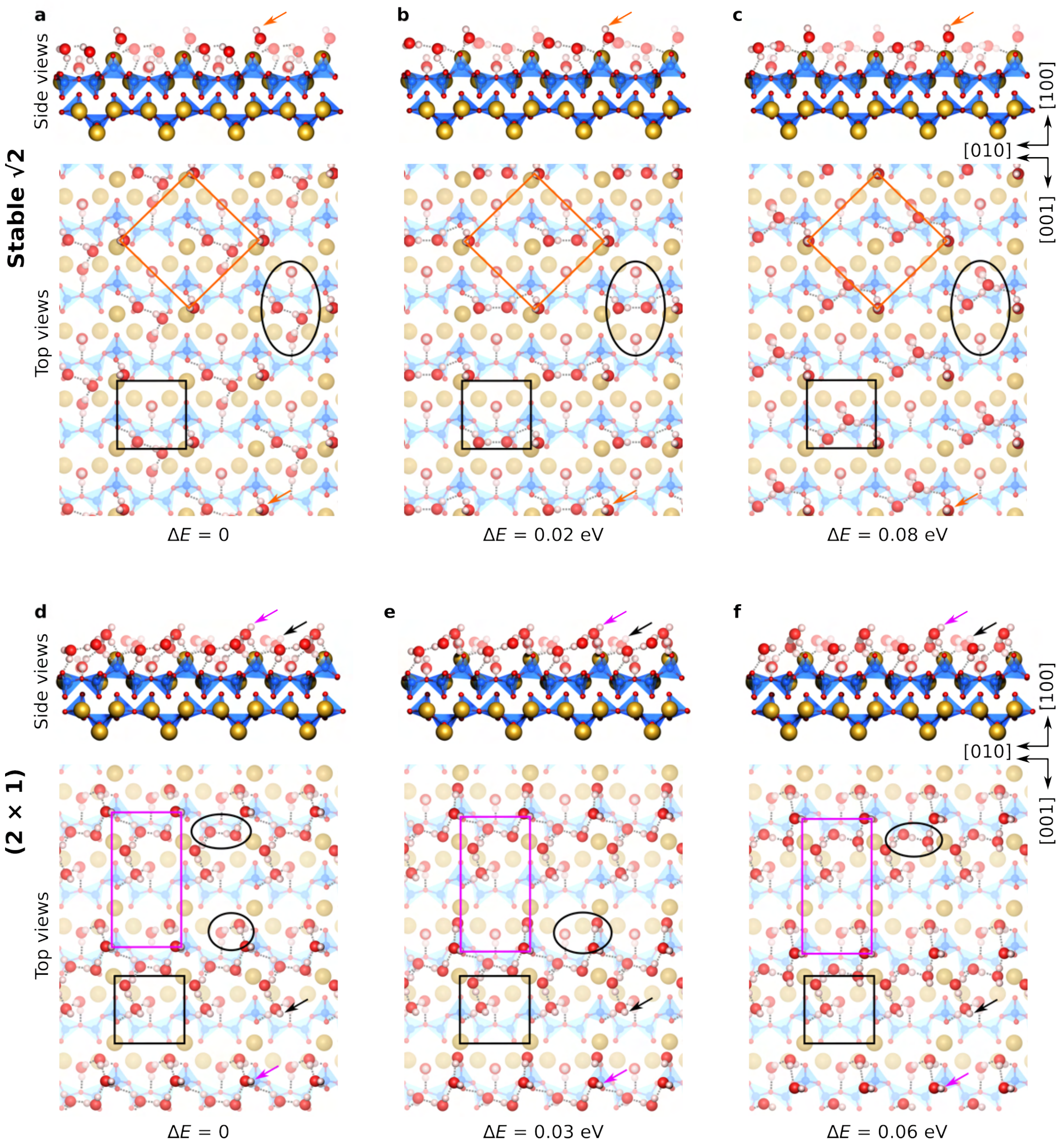

Figure S7: Comparison of energetically similar water configurations for the "stable √2" and (2 × 1) structures. (a) Lowest-energy model and (b, c) two low-energy models for a coverage of 2.5 H_2O per (1 × 1) unit cell (black rectangle) in a (√2 × √2)R45° cell (orange rectangle), aiming to model the "stable √2" pattern observed experimentally. Orange arrows indicate the most protruding H_2O molecule. (d) Lowest-energy model and (e, f) two low-energy models for a coverage of 4 H_2O per (1 × 1) unit cell in a (2 × 1) cell (magenta rectangle). Magenta and black arrows indicate the most protruding and the second-most protruding H_2O molecules, respectively. Black ovals mark molecules arranged in distinct configurations among the different models. Because these molecules do not protrude from the surface, they remain undetectable in constant-height nc-AFM measurements. Relative energy differences per unit cell (ΔE) are shown below the corresponding models. Since these differences (<0.1 eV per unit cell) lie within the typical accuracy limits of DFT for H-bonded systems,[4] these configurations are degenerate within the error bars and may coexist in experiments at finite temperatures.

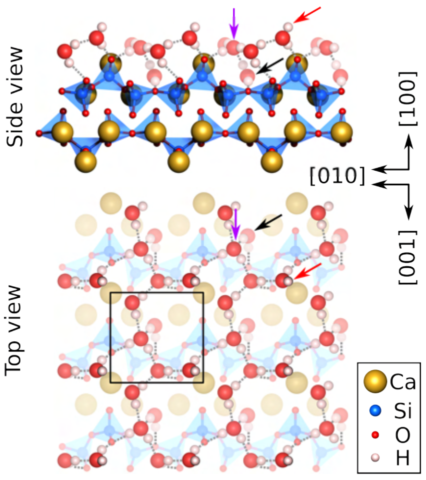

Figure S8: Lowest-energy model with a coverage of five H_2O per (1 × 1) unit cell. Water molecules are arranged into an extended H-bonded network (marked by gray dotted lines). Although the initial adsorption geometries were generated through unbiased random sampling, the most favorable model exhibits one protruding H_2O (red arrow) per unit cell bound to the Ca and H-bound to the apical oxygen of a surface silica tetrahedron. This suggests that this surface site remains active during water adsorption for all coverages above 1 ML, where not all H_2O can be accommodated as nested water. In contrast to structures at lower coverage, a second H_2O (purple arrow) donates a H-bond to the same apical oxygen. This second molecule adopts a horizontal orientation and fourfold coordination: donating two H-bonds (to the apical surface oxygen and a neighboring H_2O) and accepting two (from a planar H_2O and the nested H_2O). Similar to the (2 × 1) structure (see Figure 5f), the nested water (black arrow) is involved in the H-bonded network and remains strongly bound to its site, indicating a preference for this configuration even at the high-coverage limit. The differential adsorption energy per water molecule is shown in Figure 5d.

	$CaSiO_3$	a (rel. error)	b (rel. error)	c (rel. error)	α (rel. error)	β (rel. error)	γ (rel. error)	Cell volume (rel. error)	Bulk modulus (rel. error)	Band gap (rel. error)
**Experiment (RT)**		7.93 Å	7.32 Å	7.07 Å	90.1°	95.2°	103.4°	396.9 Å^3	102.0 GPa	5.45 eV
**GGA**	**PBE**	8.03 Å (+1.3%)	7.40 Å (+1.1%)	7.16 Å (+1.3%)	90.1° (+0.0%)	95.5° (+0.3%)	103.5° (+0.0%)	411.5 Å^3 (+3.7%)	91.4 GPa (–10.4%)	4.86 eV (–10.9%)
	**PBE-D3**	7.95 Å (+0.4%)	7.35 Å (+0.4%)	7.09 Å (+0.4%)	90.0° (+0.0%)	95.4° (+0.1%)	103.4° (+0.0%)	401.3 Å^3 (+1.1%)	93.8 GPa (–8.1%)	4.99 eV (–8.5%)
**metaGGA**	**r²SCAN**	7.92 Å (–0.1%)	7.31 Å (–0.1%)	7.07 Å (+0.1%)	90.0° (+0.0%)	95.3° (+0.1%)	103.4° (+0.0%)	396.6 Å^3 (–0.1%)	98.8 GPa (–3.1%)	5.60 eV (+2.8%)
	**r²SCAN-D3**	7.88 Å (–0.5%)	7.29 Å (–0.4%)	7.04 Å (–0.3%)	90.0° (+0.0%)	95.2° (+0.0%)	103.4° (+0.0%)	392.2 Å^3 (–1.2%)	100.8 GPa (–1.2%)	5.65 eV (+3.7%)
	**r²SCAN+rVV10**	7.89 Å (–0.5%)	7.30 Å (–0.2%)	7.04 Å (–0.3%)	90.0° (+0.0%)	95.1° (–0.1%)	103.4° (+0.0%)	393.1 Å^3 (–1.0%)	100.8 GPa (–1.2%)	5.54 eV (+1.6%)

Table T1: Experimental and calculated bulk properties of wollastonite. Lattice parameters (a, b, c, α, β, γ), unit cell volume, bulk modulus, and band gap obtained for different exchange–correlation functionals compared with experimental data. Experimental unit cell parameters were derived from XRD analysis[5] at room temperature (RT), while the bulk modulus and band gap values were taken from Refs. 6,7, respectively. Brackets indicate relative errors in percent with respect to the experimental values. The PBE-D3 and r²SCAN+rVV10 functionals provide the most accurate description of the bulk phase (lattice parameters, bulk modulus, and band gap) within the GGA and metaGGA families, respectively.

System	Surface unit cell dimensions	Surface unit cell area	Water coverage per surface unit cell	Surface water density	Experimentally observed structures
**Bulk ice Ih**	7.79 Å × 7.79 Å	52.6 Å^2	6 H_2O	11.4 H_2O/nm^2	-
**Silver iodide (AgI)**	9.18 Å × 9.18 Å	73.0 Å^2	8 H_2O	11.0 H_2O/nm^2	Epitaxial ice Ih layer
**Wollastonite ($CaSiO_3$)**	7.07 Å × 7.32 Å	51.8 Å^2	2.5 H_2O	4.8 H_2O/nm^2	Stable √2
			4 H_2O	7.7 H_2O/nm^2	(2 × 1)
			5 H_2O	9.7 H_2O/nm^2	3D clusters

Table T2: Surface water density for bulk ice Ih, silver iodide, and wollastonite. Comparison of surface unit cell dimensions, areas, and corresponding surface water densities for bulk ice Ih,[8] (2 × 2)-reconstructed AgI(0001) (a perfect ice nucleator), and (100)-oriented wollastonite ($CaSiO_3$). Silver iodide promotes the growth of an epitaxial ice layer (11.0 H_2O/nm^2).[9] Conversely, while a coverage of 5 H_2O per unit cell on wollastonite yields a similar surface water density (9.7 H_2O/nm^2), the rectangular and corrugated substrate geometry precludes planar epitaxy, driving the formation of 3D clusters.

References

1. G. Franceschi, A. Conti, L. Lezuo, R. Abart, F. Mittendorfer, M. Schmid and U. Diebold, *J. Phys. Chem. Lett.*, 2024, **15**, 15–22.
2. R. C. Longo, K. Cho, P. Brüner, A. Welle, A. Gerdes and P. Thissen, *ACS Appl. Mater. Interfaces*, 2015, **7**, 4706–4712.
3. P. Thissen, C. Natzeck, N. Giraudo, P. Weidler and C. Wöll, *Chem. - Eur. J.*, 2018, **24**, 8603–8608.
4. M. J. Gillan, D. Alfè and A. Michaelides, *J. Chem. Phys.*, 2016, **144**, 130901.
5. A. Conti, L. Lezuo, A. Hoheneder, E. Vaníčková, D. A. Aloi, A. Steiger-Thirsfeld, D. Heuser, R. Abart, F. Mittendorfer, M. Schmid, U. Diebold and G. Franceschi, *ACS Nano,* 2026, **20**, 10456–10465.
6. N. I. Demidenko and A. P. Stetsovskii, *Glass Ceram.*, 2003, **60**, 217–218.
7. H. Nagabhushana, B. M. Nagabhushana, M. Kumar, H. B. Premkumar, C. Shivakumara and R. P. S. Chakradhar, *Philos. Mag.*, 2010, **90**, 3567–3579.
8. K. Röttger, A. Endriss, J. Ihringer, S. Doyle and W. F. Kuhs, *Acta Cryst. B*, 1994, **50**, 644–648.
9. J. I. Hütner, A. Conti, D. Kugler, F. Sabath, K. N. Dreier, H.-G. Stammler, F. Mittendorfer, A. Kühnle, M. Schmid, U. Diebold and J. Balajka, *Sci. Adv.*, 2025, **11**, eaea2378.
